# Comparison of the effects of open- and closed-skill exercise on cognition and peripheral proteins: A cross-sectional study

**DOI:** 10.1371/journal.pone.0251907

**Published:** 2021-06-04

**Authors:** Evrim Gökçe, Emel Güneş, Fikret Arı, Serhat Hayme, Erhan Nalçacı

**Affiliations:** 1 Ankara City Hospital, Sports Rehabilitation Laboratory, Ankara, Turkey; 2 Department of Physiology, Ankara University School of Medicine, Ankara, Turkey; 3 Electrical and Electronics Engineering, Ankara University Faculty of Engineering, Ankara, Turkey; 4 Department of Biostatistics, Ankara University School of Medicine, Ankara, Turkey; Iwate Medical University, JAPAN

## Abstract

Previous research indicates that different exercise modes might create different effects on cognition and peripheral protein signals. This study aimed to compare the effects of long-term participation in an open and closed-skill exercise on cognitive functions and Brain-derived neurotrophic factor and Cathepsin B levels. 18 fencers, 18 swimmers, 18 sedentary controls between 18–25 years old participated in the study. Participants performed visuospatial working memory, verbal fluency and selective attention tasks. Blood samples were tested for Brain-derived neurotrophic factor and Cathepsin B using ELISA. The results showed that fencers performed superiorly on some part of visuospatial working memory, verbal fluency, and selective attention tasks than swimmers and sedentary controls. Athlete groups showed higher scores on some subtests of visuospatial working memory and selective attention tasks than sedentary controls. The basal serum Brain-derived neurotrophic factor level was not significant between the groups, but Cathepsin B was higher in fencers than swimmers and sedentary controls. The peripheric protein signal response to acute exercise was significantly higher in athletes, particularly in the open-skill group for Cathepsin B. Our research provided noteworthy results that more cognitively challenging exercise may provide more benefits for some aspects of cognition. Since our findings suggest that open-skill exercise improves specific types of executive-control functioning, this exercise mode might be included in training programs to support cognition and prevent cognitive impairment.

## Introduction

In recent years, the literature has been increasing, highlighting the beneficial effects of regular exercise on cognitive functions [1]. Cognitive functions refer to mental processes of obtaining and understanding knowledge, including memory, attention, visual and spatial processing, language, executive functions [2].

Executive function refers to higher-order cognitive processes that aid in the monitoring and controlling thought and action and plays a crucial role in daily life. It contains working memory, cognitive flexibility, reasoning, planning, problem-solving, and inhibitory control [3]. Many cognitive functions, such as processing speed, visuospatial function, and control processes (e.g., inhibition, planning, scheduling, and working memory) are reported to improve via physical exercise, especially in older adults [1, 4].

However, there is an increasing literature that seems to support the view that different types of physical exercise can affect the brain in different ways. Continuous observations showed that exercise mode is an important factor that affects exercise’s impact on cognition. Different physical exercise modes with different cognitive loads and motor-coordination skills have been related to neurocognitive improvement levels [5].

Open-skill exercise and closed-skill exercise classification is based on the performance’s consistency and predictability [6]. The open-skill exercise (e.g., volleyball, badminton, and fencing) requires behavioral and motor adaptation to respond to external-paced, unpredictable stimuli and needs more cognitive resources than closed-skill exercise (e.g., swimming and walking). Closed-skill exercise is performed in a relatively stable environment and tends to be self-paced. The present study aimed to distinguish the relationship between exercise mode and cognitive improvement based on open and closed-skill exercise classification.

Some studies demonstrate open-skill exercise showed better visuospatial attention [7], inhibitory control [8], problem-solving [9], cognitive flexibility [10], and lower switch cost of reaction time scores [11] than closed-skill exercise.

A cross-sectional study showed that regular open-skill exercise promotes executive functions, likely due to various sport training characteristics that involve more complex cognitive processes [12]. Similarly, open-skill exercise had been demonstrated to improve executive network efficiency compared to closed-skill exercise [13]. This improvement in the attentional system component has been interpreted as a result of the open skill exercise combining physical exercise and cognitive training at the same time [14]. A systematic review has reported that open-skill exercise seems more effective for improving some aspects of cognitive function than closed-skill exercise [4]. However, there is also a study that open-skill exercise shows no superiority over closed-skill exercise to improve cognitive skills [15].

It has been reported that different kinds of exercise seem to have specific effects on neurocognitive performance due to the differences in the secretion of some biomarkers in the neurochemical system [16]. Brain-Derived Neurotrophic Factor (BDNF) and Cathepsin B (CTSB) are peripheral factors considered modulators of physical exercise and cognition relation. BDNF is a key protein that regulates neuronal development, survival, and plasticity in mammals [17]. It is expressed in neuronal and non-neuronal tissues and stored peripherally in platelets [18, 19]. Although the brain contributes to 75% of BDNF synthesis under normal conditions, it is also synthesized in skeletal muscle. BDNF is found throughout the nervous system but is primarily concentrated in the cortex and hippocampus. BDNF has been hypothesized to be a potential underlying mechanism for the effects of exercise on cognition [17–19].

However, CTSB is relatively less studied compared to BDNF in exercise literature. CTSB is a papain superfamily member and is considered vital in neuroprotective lysosomal activation, neuronal survival [20]. It has significant anti-amyloidogenic activity [21]. In response to exercise, muscles release CTSB into the circulation [22]. CTSB is shown as a muscle secretory factor that is important for the cognitive and neurogenic benefits of exercise and, it has been suggested that recombinant CTSB application enhanced expression of BDNF in adult hippocampal progenitor cells [20]. Both BDNF and CTSB are myokines capable of crossing the blood-brain barrier [20, 23]. Because the skeletal muscle plays a critical role in exercise, these myokines may affect neural plasticity.

The current study hypothesizes that regular open-skill exercise would elicit better cognitive functions and higher basal serum BDNF and CTSB levels than closed-skill exercise and inactivity. Further, compared with the closed-skill exercise and inactivity, the regular open-skill exercise would elicit stronger BDNF and CTSB response after an acute bout of aerobic exercise including a cognitive task.

## Material-method

The ethics committee of Ankara University Faculty of Medicine approved this study with number 36 at 25.10.2017. The experiments were conducted with the understanding and the written consent of each volunteer.

### Participants

Fifty-four healthy subjects between 18 and 25 years were recruited in this study. Participants were required to meet the following criteria: right-handed, normal or corrected-to-normal vision, the body mass index (BMI) less than 25. Smoking habits, a history of severe disease, cranioencephalic trauma, cognitive deficiencies, taking psychoactive drugs were exclusion criteria. Five applicants were excluded from participation in this step.

According to their exercise modes, eligible participants were assigned to three groups (open-skill = fencing, closed-skill = swimming, control = sedentary). The sedentary control group who had not been involved in regular physical training of any sport was recruited through community announcements from universities in Ankara. Athletes were recruited from national fencing and swimming clubs. They were required to perform the same sport for at least five years. Athletes must have participated in at least one national competition over the last year.

Before the experiment, participants were required to fill in the demographic questionnaire, exercise history questionnaire, Turkish Version of Chapman and Chapman’s hand preference questionnaire [24], and The Turkish Version of the International Physical Activity Questionnaire (IPAQ) [25]. The years of education, daily habits (e.g., listening to music, reading books, playing computer games) were recorded to control bias between sedentary and exercise groups. The sport practice years average, the frequency, duration, and intensity of their exercise practice were recorded for athletes. Athletes reported participating in training five times per week for at least 1 hour. The sedentary control group reported inactivity or low activity levels in IPAQ and no regular exercise. Women were recruited during the luteal phase of the menstrual cycle. An outline of the participants’ demographics and physical characteristics are summarized in Table 1.

**Table 1:** Participant characteristics and physical fitness indices across the three groups (mean ± standard deviation).

	Sedentary	Fencing	Swimming
Demographics			
**Female/Male (n)**	11/7	9/9	9/9
**Age (years)**	22.33 (1.94)	20.44 (1.85)	21 (1.97)
Education (years)*	14.89 (1.32)^F^	13.89 (1.07)	14.06 (1.16)
**Training history (years)**	-	8.27 (2.13)	8.22 (2.66)
**Hand preference score**	14.06 (1.19)	13.44 (1.65)	13.11 (1.77)
Reading book (unit/year)*	28.00 (19)^F^	13.88 (16.33)	13.55 (7.52)
**Listening to music (hour/day)**	1.76 (1.30)	1.94 (1.78)	2.50 (2.17)
**Using internet (hour/day)**	4.61 (2.06)	4.30 (2.21)	3.66 (1.32)
Physical fitness			
**BMI (kg/m**^**2**^**)**	23.36 (3.52)	23.90 (1.02)	24.23 (0.97)
**VO**_**2max**_ **(ml·kg**^**-1**^**·min**^**-1**^)**	45.36 (6.57)	57.05 (7.86)^C^	54.60 (7.67)^C^
Muscle mass (kg)*	46.33 (9.05)	57.61 (13.49)^C^	54.77 (11.99)^C^
Fat mass (%)*	22.60 (6.38)	17.12 (3.93)^C^	16.58 (7.58)^C^
Basal metabolism (kcal)**	2517.38(853.32)	3801.94(1019.65)^C^	3355.77(673.03)^C^
IPAQ score (met)**	429.17(122.3)	7526.91(6252.00)^C^	7317.14(4352.00)^C^

**Note**. F, S, C, indicate the denoted value is significantly different from that of the fencing, swimming, or control group in the same row. Asterisks indicate significance [**p < 0.001; *p < 0.05]. Data are presented as mean ± SD.

Participants were instructed to avoid strenuous exercise for 24 h before the experiment, while food, caffeine, and alcohol intake were also prohibited for eight hours before each session.

All subjects were informed verbally and in writing about the study’s nature, including all potential risks, and all signed the informed consent forms before the experiment. The experiment was compliant with the ethical principles of the Declaration of Helsinki. The ethics committee of approved this study.

**Fig 1:**
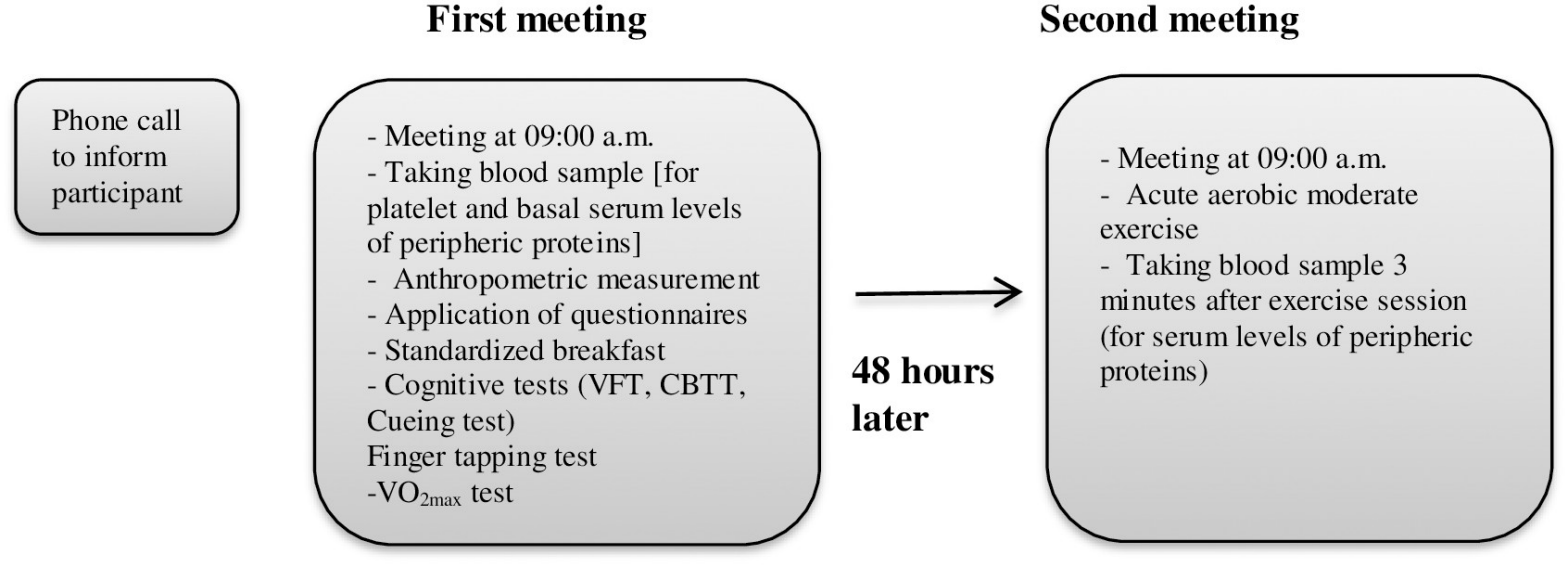
The diagram of the experiment procedure.

The diagram of the experimental procedure is given in Fig 1.

### VO_2max_ assessment

Cardiorespiratory fitness assessment was measured by the Bruce Protocol, which is a maximal graded exercise test on a motorized treadmill [26]. During this test, both the speed and slope increased every 3 minutes until participants were exhausted and/or reached age-predicted maximum heart rate (220-age).

The formula for calculating VO_2max_ (ml·kg^-1^·min^-1^) was as follows:

$$\text{For}\;\text{Men}\;{\text{VO}}_{2\text{max}}=14.8–(1.379\;\text{x}\;\text{T)}+(0.451\;\text{x}\;{\text{T}}^{\text{2}}\text{)}–(0.012\;\text{x}\;{\text{T}}^{\text{3}}\text{)}$$

$$\text{For}\;\text{Women}\;{\text{VO}}_{2\text{max}}=4.38\;\text{x}\;\text{T}\;\text{-}\;\text{3}.\text{9}$$

T = Total time on the treadmill measured as a fraction of a minute.

### Acute exercise protocol

In the acute exercise sessions, participants completed 40 minutes of running on a treadmill. The exercise intensity was planned on the heart rate reserve (HRR) [27]. The target HR was calculated as follows:

TargetHR=(HRR×percentageintensitydesired)+RestingHR.

The desired exercise intensity range was 50%–60% of HRR. HR was monitored by a chest strap heart rate monitor (Polar H10 Heart Rate Sensor).

### Cognitive assessment

All computerized cognitive tests were developed under the MATLAB (Mathworks^®^. Version R2017a) computer programming language.

#### Corsi’s Block Tapping Test

The modified Corsi’s Block Tapping Test (CBTT) is a computerized version of the block tapping test that measures visuospatial short-term memory [28]. Participants were seated in front of a monitor at a distance of 70 cm. There was a familiarization test. For each trial, the cubes appeared on the computer screen in a varying sequence. After four seconds of the presentation, the screen changed, and the participants were asked to place the correct position of the cubes by clicking appropriate places on the empty grid with a mouse.

The task began with a three-box sequence and continued until a maximum of nine-box. The test terminated when the participant cannot remember the sequence for two consecutive trials at any one level. The test was randomly practiced with right and left hands for each participant. On 17 trials, a total of 112 objects (42 in the right hemispace, 42 in the left hemispace, and 18 in the middle) were presented. Accuracy (percent of correct responses) and neglect scores were calculated for each hemispace separately. The neglect score was calculated by subtracting the number of placed cubes by the participant (regardless of correctness) from the number of cubes presented.

#### Spatial cueing test

We used a spatial orienting task with an endogenous (an arrow) and exogenous visual cue (brightness) that directs the attention to one of two locations within the visual field to explore selective attention [29]. All stimuli were presented on a computer screen viewed from a distance of 57 cm. Participants were instructed to press the right or left arrow button on the keyboard as soon as they detected using their preferred hand. Participants were presented with a fixation point and two boxes located to the fixation’s left and right. The cue was presented centrally for the endogen task and peripherally for the exogenous task. The target was presented in the left or right boxes. For cueing, an endogenous cue consisting of an arrow pointing in one of the two directions was presented at fixation. A total of 120 trials were presented for each task with a valid cue (%60), invalid cue (%30), and no cue (%10). Accuracy (number of correct responses) and reaction time (RT) (ms) were recorded to analyze.

#### Verbal fluency test

Verbal Fluency Test (VFT) was assessed in two subheads: Letter fluency and category fluency [30]. Participants were asked to name as many words as possible in 1 minute with specified letters (A-S-K) and categories (animal-fruit-supermarket products). Total accuracy and error score were calculated for letter and category tasks. Accuracy score is the number of words counted following the specified rules. Words that did not comply with the specified rules or duplications were recorded as error score.

### Biochemical analysis

#### Sampling

Venous blood samples were collected from the antecubital vein in tubes containing EDTA as an anticoagulant to obtain serum between 9:00 and 10:00 a.m. after overnight fasting. For platelet analysis, blood samples were assessed in between 2 and 6 hours.

Peripheral protein analysis was performed at both the basal level and after acute exercise. For post-exercise measurements, blood samples were collected 3 minutes after the exercise session ended. The blood was allowed to coagulate for 120 minutes at room temperature. The serum was centrifuged at 1.000 × g. 20 minutes, and stored at −80°C until analyses.

#### Peripheric protein analysis

Venous blood samples were obtained two times: The first day of the experiment for the basal level and the second day of the experiment 3 minutes after the acute moderate exercise.

Serum Cathepsin B and BDNF levels were measured using an ELISA Kit (MyBioSource Cathepsin B. Catalog no: MBS9305224; MyBioSource BDNF. Catalog no: MBS2515054) following the manufacturer’s instructions.

### Statistical analysis

Statistical analysis was performed using SPSS 20.0 for Windows. Descriptive statistics are given as "mean ± standard deviation" for variables with normal distribution and as "median (min; max)" for variables with non-normal distribution. ANOVA for mean values and Kruskal Wallis test for median values evaluated the significance of the difference between groups. Post-hoc comparisons were performed by Tukey and Dunn-Bonferroni analyses. The significance level was set at p < 0.05. We interpreted the magnitude of the effect size as (>0.20 small; >0.50 moderate; >0.80 large) [31]. Tables 2 and 3 reported results of all measures.

**Table 2:** Results of cognitive tests by group.

		Fencing	Swimming	Control
**Corsi’s Block Tapping Test**	**Right hand**			
	Right hemispace accuracy	77 (53;94)	78 (61;94)	76 (53;94)
	Left hemispace accuracy**	94 (73;100)^S-C^	84 (56;100)	78 (48;100)
	Total accuracy	85 (67;97)	82 (61;97)	78 (49;95)
	Neglect	13 (3;25)	17 (3;39)	22 (5;54)
	**Left hand**			
	Right hemispace accuracy	67 (41;89)	67 (58;74)	62 (48;74)
	Left hemispace accuracy*	91 (43;100)^C^	93 (78;97)^C^	85 (65;97)
	Total accuracy	87 (67;97)	88 (71;95)	82 (64;95)
	Neglect*	12 (3;33) ^C^	11 (5;29)^C^	18 (5;36)
**Spatial Cueing Test**	**Endogenous**			
	*Valid*			
	Accuracy (R)	33 (32;35)	33 (31;35)	33 (30;35)
	Accuracy (L)	33 (32;35)	33 (31;35)	32 (30;35)
	RT (R)	266 (189;310)	275 (102;328)	296 (265;320)
	RT (L)	280 (195;317)	276 (157;318)	293 (319;269)
	*Invalid*			
	Accuracy (R)**	15 (14;18)^C^	15 (14;17)^C^	14 (13;16)
	Accuracy (L)**	15 (14;17)^C^	15 (14;17)^C^	14 (12;16)
	RT (R)*	282 (205;316)^C^	291 (253;329)^C^	306 (283;325)
	RT (L)	290 (205;321)	289 (173;450)	298 (279;317)
	**Exogenous** Yüzme Sedanter p değeri			
	*Valid*			
	Accuracy (R)	33 (31;36)	32 (31;35)	32 (30;34)
	Accuracy (L)	33 (32;35)	32 (30;34)	32 (31;34)
	RT (R)*	254 (187;289)^S-C^	275 (213;307)	282 (245;302)
	RT (L)*	263 (198;291)^C^	274 (223;297)	283 (250;305)
	*Invalid*			
	Accuracy (R)	14 (14;17)	14 (13;17)	14 (12;16)
	Accuracy (L)	14 (12;16)	14 (13;16)	14 (12;16)
	RT (R)	279 (200;300)	287 (233;317)	290 (255;319)
	RT (L)	283 (228;301)	294 (253;319)	293 (258;317)
**Verbal Fluency Test**	**Letter Fluency**			
	Accuracy	48.63 (28;67)	40.33 (19;54)	40.11 (31;60)
	Error**	0.11 (0;1)^C^	0.39(0;1) ^C^	1.89 (0;4)
	**Category Fluency**			
	Accuracy	61.67 (48;81)	56.72 (41;66)	60.50 (46;86)
	Error*	0.06 (0;1) ^C.S^	0.50 (0;3)	0.83 (0;2)

**Note**. F, S, C, indicate the denoted value is significantly different from that of the fencing, swimming, or control group in the same row. Asterisks indicate significance (**p < 0.001; *p < 0.05). Data are presented as the median (minimum and maximum values). R: Right. L: Left. RT: reaction time. F: Fencing. S: Swimming. C: Control. Accuracy score is presented as the number of correct responses for Spatial Cueing Test and VFT. The error score is presented as the number of incorrect responses. Accuracy and neglect scores are presented as the percent for CBTT. RT is presented as ms.

**Table 3:** Results of peripheral proteins by group.

Basal-BDNF	Fencing	Swimming	Control
	23.37 (8.13)	21.25 (3.45)	19.35(4.70)
Post-BDNF **	28.62 (13.95;43.45)^C^	26.92 (18.32;39.33) ^C^	18.03 (12.71;23.92)
ΔBDNF (%)**	22.50 (3.62)^C^	26.58 (4.40) ^C^	6.82 (5.07)
Basal-CTSB**	11.32 (6.28;25.61)^C-S^	6.13 (1.00;19.76)	6.02 (2.57;13.10)
Post-CTSB **	18.16 (4.91;85;39) ^C-S^	7.64 (2.79;22.36)	7.09 (3.10;11.64)
ΔCTSB (%)*	53.58 (4.07) ^C-S^	24.63 (3.37)	17.77 (3.38)

**Note**. F. S. C. indicate the denoted value is significantly different from that of the fencing, swimming, or control group in the same row. Asterisks indicate significance (**p < 0.001; *p < 0.05). Normally distributed data are presented as mean ± SD. Non-parametric data are presented as the median (minimum and maximum values). BDNF and CTSB levels are presented as ng/ml. Basal-Post BDNF, Basal-Post CTSB differences are presented as Δ (%).

## Results

### Demographic and physical characteristics of participants

Demographic variables including age, hand preference score, listening to music/using internet span did not differ between the groups. Education year was significantly different between the groups, *F*(2,51) = 3.634, *p* = 0.033, sedentary controls had longer education years than fencers (*p* = 0.039). The number of books read annually was significantly different between the groups, *H*(2) = 6.816, *p* = 0.033; sedentary controls read more books than fencers (*p* = 0.021).

As to physical characteristics, BMI did not differ between the groups. *F*(2,51) = 0.274, *p* = 0.761. VO_2max_ was significantly different among the groups, *F*(2,51) = 12.520, *p* < 0.001. Fencers (*p* < 0.001) and swimmers (*p* = 0.001) had higher VO_2max_ levels than sedentary controls. Muscle mass was significantly different among the groups, *F*(2,51) = 4.556, *p* = 0.015. Fencers (*p* = 0.016) and swimmers (*p* = 0.050) had more muscle mass than sedentary controls. Fat mass was significantly different among the groups, *F*(2,51) = 5.265, *p* = 0.008. Fencers (*p* = 0.030) and swimmers (*p* = 0.015) had lower fat mass than sedentary controls.

Basal metabolism was significantly different among the groups, *F*(2,51) = 10.342, *p* < 0.001. Fencers (*p* < 0.001) and swimmers (*p* = 0.015) had higher basal metabolism than sedentary controls. IPAQ score was significantly different among the groups, F(2,51) = 4.792, *p* < 0.012. Fencers (*p* < 0.001) and swimmers (*p* < 0.001) had higher IPAQ scores than sedentary controls.

There was no significant difference between athlete groups, including VO_2max_, muscle mass, fat mass, basal metabolism, and IPAQ score.

### Corsi’s Block Tapping Test

In the right-handed test, left hemispace accuracy was significantly different among the groups, *H*(2) = 15.336, *p* < 0.001, *η*^*2*^ = 0.28. Fencers showed higher scores than swimmers (*p* = 0.034, *r* = -0.41) and sedentary controls (*p* < 0.001, *r* = -0.65).

In the left-handed test, the left hemispace accuracy score was significantly different among the groups, *H*(2) = 12.318, *p* = 0.002, *η*^*2*^ = 0.23. Both fencers (*p* = 0.017, *r* = -0.46) and swimmers (*p* = 0.003, *r* = -0.53) showed higher accuracy scores than the sedentary control group in the left hemispace. The neglect score was significantly different among the groups, *H*(2) = 6.707, *p* < 0.035, *η*^*2*^ = 0.13. Sedentary control group had higher neglect scores than fencers (*p* = 0.049, *r* = -0.4) and swimmers (*p* = 0.049, *r* = -0.4).

### Spatial cueing test

In the endogenous cueing test invalid condition, when the target was on the right, RT was significantly different among the groups, *H*(2) = 11.687, *p* = 0.003, *η*^*2*^ = 0.22. Fencers (*p* = 0.005, *r* = -0.53) and swimmers (*p* = 0.021, *r* = -0.43) had significantly shorter RTs than sedentary control group.

In the invalid condition, when the target was in the right, accuracy score was different among the groups, *H*(2) = 19.563, *p* < 0.001, *η*^*2*^ = 0.36. Fencers (*p* < 0.001, *r* = -0.65) and swimmers (*p* = 0.005, *r* = -0.58) were more accurate than sedentary control group. When the target was in the left, accuracy score was different among the groups, *H*(2) = 20.304, *p* < 0.001, *η*^*2*^ = 0.38. Fencers (*p* < 0.001, *r* = -0.64) and swimmers (*p* < 0.001, *r* = -0.64) were more accurate than sedentary controls.

In the exogenous cueing test valid condition when the target was on the right, RT was significantly different among the groups, *H*(2) = 10.118, *p* = 0.006, *η*^*2*^ = 0.19. Fencers had shorter RTs than swimmers (*p* = 0.022, *r* = -0.38) and the sedentary controls (*p* = 0.006, *r* = -0.46).

When the target was on the left, RT was significantly different among the groups, *H*(2) = 7.776, *p* = 0.020, *η*^*2*^ = 0.14. Fencers had shorter RTs than the sedentary controls (*p* = 0.017, *r* = -0.43).

### Verbal fluency test

Letter error score was significantly different among the groups, *H*(2) = 19.296, *p* < 0.001, *η*^*2*^ = 0.36. Fencers (*p* < 0.001, *r* = -0.64) and swimmers (*p* = 0.002, *r* = -0.51) made less errors than sedentary controls. Category error score was significantly different among the groups, *H*(2) = 9.195, *p* = 0.010, *η*^*2*^ = 0.17. Fencers made less errors than swimmers (*p* = 0.035, *r* = -0.35) and sedentary controls (*p* = 0.003, *r* = -0.50).

### Brain-derived neurotrophic factor

Basal serum BDNF levels did not show a significant difference between the groups. After acute exercise, BDNF level was significantly different among the groups, *H*(2) = 18.601, *p* < 0.001, *η*^*2*^ = 0.35. Fencers (*p* = 0.001, *r* = -0.53) and swimmers (*p* < 0.001, *r* = -0.70) had higher BDNF levels compared to sedentary controls. ΔBDNF showed difference among the groups, *F*(2, 51) = 15.491, *p* < 0.001, *η*^*2*^ = 0.38. Fencers (*p* < 0.001) and swimmers (*p* < 0.001) showed higher change compared to sedentary controls. BDNF increased in athlete groups after exercise, although decreased in sedentary controls.

### Cathepsin B

Basal serum CTSB level was significantly different between the groups, *H*(2) = 18.271, *p* = 0.000, *η*^*2*^ = 0.34. Fencers had higher levels than swimmers (*p* = 0.003, *r* = -0.48) and sedentary controls (*p* < 0.001, *r* = -0.72).

After acute exercise, CTSB level was significantly different among the groups, *H*(2) = 16.566, *p* < 0.001, *η*^*2*^ = 0.31. Fencers had higher levels than swimmers (*p* = 0.001, *r* = -0.53) and sedentary controls (*p* < 0.001, *r* = -0.61). ΔCTSB showed difference among the groups, *F*(2, 51) = 5.908, *p* = 0.005, *η*^*2*^ = 0.19. Fencers showed higher change compared to swimmers (*p* = 0.043) and sedentary controls (*p* = 0.027).

## Discussion

### Corsi’s Block Tapping Test

CBTT is an executive function test that evaluates spatial perception associated with working memory and requires visual-spatial storage and active processing. A study examining the effects of open and closed-skill exercises on visual-spatial working memory scores reported that open-skill exercise athletes achieved higher scores than closed-skill exercise athletes and sedentary and did not find a difference between closed-skill exercise athletes and sedentary ones [32]. In line with the literature, we found that fencers performed higher accuracy scores in the left hemispace than swimmers and the control group in the right-handed task. Fencers’ higher left hemispace accuracy scores may suggest that open-skill exercise increases the asymmetry of functions, lateralization of visuospatial working memory.

We found higher left hemispace accuracy and lower neglect scores in the left-handed task in exercise groups indicating the overall enhancing effect of the exercise on visual-spatial working memory, and no significant difference was observed between the two exercise groups. However, a study comparing the open and closed-skill exercises found a significant difference in working memory tasks only for closed-skill exercise [33]. On the other hand, a study that assesses visuospatial working memory with CBTT pointed out that athletes who play basketball for ten years did not differ from inactive people [34].

### Spatial cueing test

It has been demonstrated that shorter RTs for fencing and water polo athletes than swimmers and sedentary controls in the exogenous cueing test [35]. In line with the literature, we demonstrated that RT was shorter in fencers than swimmers and sedentary controls when the target was in the right in valid exogenous cueing test trial. It may be related that the exogenously directed attention can be sustained for a shorter time. On the contrary, an EEG study comparing open and closed-skill exercises have not significantly differed in visuospatial attention data between the groups [15]. This contradiction might be originated fencing demands more alertness, fast response, and precision due to the recurrent reduction of distance between the opponents than the open-skill sport type the above-mentioned study chose.

Due to spatial attention is oriented faster when directed exogenously, endogenous attention can be sustained for longer periods than exogenous attention. Our non-significant results between fencer and swimmer groups, especially in RTs in endogenous attention test that may be related to this situation. The shorter reaction time in the exercise groups while the target was on the right in the invalid endogenous cueing test can be interpreted as the right hemisphere is more sensitive to the distracting cue. Because the right hemisphere specializes in attention-related functions, exercise groups were better at inhibiting the distracting cue. Elite athletes are more successful in visuospatial attention tasks than sedentary controls [36]. It was showed that three times a week for three years, Tai-Chi exercise improved attention and volleyball players have a shorter reaction time in attention tasks than sedentary controls [37]. Our results aligned with the literature above and indicated that the exercise groups were more successful than the sedentary controls for accuracy and reaction time.

### Verbal fluency test

The verbal fluency tests rely on both intact memory stores and simple access to the information. In addition to assessing memory and knowledge, verbal fluency may also link other cognitive processes, including reasoning to generate category examples, searching subcategories while maintaining a cognitive set of the overarching category, working memory to inhibit previous responses, and inhibition of non-category items.

This study observed no significant difference between the groups in terms of accuracy scores of letter and category tasks. However, fencers made fewer mistakes than swimmers and sedentary controls in category tasks. This finding indicates that working memory and inhibitory control [for remembering counted words previously and avoid repeating them] are more developed in open-skill exercise. Additionally, fewer error scores of exercise groups in letter fluency can be explained by the overall improving effect of the exercise on working memory and inhibition.

In previous studies, VFT has been used to evaluate the effects of exercise on neurodegeneration, psychiatric diseases, and aging; therefore, the findings emerged stronger [38, 39]. The age range of the participants in this study may be related to the slighter outcomes. More challenging test alternatives may be needed to assess verbal skills in young adults.

### Brain-derived neurotrophic factor

We did not find a significant difference of basal BDNF levels between the groups. As has been already stated by previous writers, chronic exercise and BDNF relationship is contradictory [40]. There is convincing evidence that chronic exercise training increases BDNF levels and improves memory performance among animal models; however, the findings within human studies are less clear [41]. A small cross-sectional study of 44 subjects reported an inverse correlation between BDNF and physical activity [42]. The heterogeneous findings have been explained by methodological approaches and differences in study populations like age or gender [43].

It was demonstrated that peripheral BDNF increase is transient after acute exercise [44] and returns the baseline level in 10–60 minutes [45]. Two different systematic reviews have pointed out that most of the studies prefer "immediately after" measurements as a methodological approach [43, 45]. Because the literature emphasized the importance of blood collection time in BDNF measurement after acute exercise, we standardized the blood drawing time as 3 minutes.

Moreover, there are no age or gender matches in the studies cited above, but our study matched the groups based on age, and gender is slightly different only in the sedentary control group. BDNF plasma levels are more stable in women, whereas it peaks in the morning and decreases throughout the day [46]. For this reason, we drew the first blood samples at 09:00 in the morning from all of the participants. It is widely known that serum BDNF level increases in the luteal phase in women [47]. Hence, female participants were included in the study when they’re in the luteal phase, based on the pre-experiment phone call. It has also been suggested that higher platelet counts will decrease BDNF levels in the serum, as platelets store BDNF [48]. There was no significant difference in platelet counts between the groups in our study.

Recent studies have shown that environmental enrichment promotes BDNF gene expression [49], and an acute bout of open-skill exercise increases the BDNF secretion compared to closed-skill exercise [50]. Unlike the literature reports, we observed no significant difference in BDNF response to exercise between sports groups. However, the acute exercise session that we used in this study was a closed-skill running protocol. Therefore, the fact that the BDNF response to acute exercise did not differ between athlete groups may be related to the protocol we have chosen.

It has been reported that BDNF response to exercise is related to exercise volume [intensity, duration, frequency] and individual’s exercise capacity [51]. As stated literature above, our data revealed that sports groups showed a stronger reaction to exercise. Cortisol increases in response to stressors have a negative effect on BDNF levels [52]. Considering exercise is a potent physiological stressor for sedentary individuals, decreased BDNF level after acute exercise in sedentary controls may be related to this.

### Cathepsin B

There are conflicting results regarding exercise and CTSB relationship in the literature. One study that has a small sample size indicated no change in CTSB after eight weeks of exercise [53], another study stated a decrease in the basal plasma CTSB levels after long-term exercise in men [54], and lastly, a study showed there was an increase in CTSB levels after four months exercise intervention [20]. In this study, fencers had higher basal/post-exercise serum levels and ΔCTSB than swimmers and sedentary controls. Both long-term participation and acute exercise effect on basal CTSB level were only limited to the open-skill exercise group. This result suggests that CTSB may be a more responsive peripheral protein to exercise than BDNF.

### General discussion

Research comparing the effects of open vs. closed-skill exercises on cognition is quite limited. Observational studies have supported that open-skill exercise issues better cognitive performance [8, 55].

Participants need to adapt to a constantly changing environment during open-skill exercise compared to closed-skill exercise. More cognitively challenging sport features, including processing speed, cognitive flexibility, and inhibitory control task, may be effective in further developing these aspects of cognitive function in open-skill exercise. The social interaction during open-skill exercise may also have a more beneficial effect on cognition. In fencing, the requirement for close monitoring of the opponent’s movements may be considered examples of this interaction.

A study comparing children’s flanker task performance based on VO_2max_ levels showed that those with higher VO_2max_ levels had higher accuracy scores and shorter RTs [56]. In line with the literature, considering the athlete groups’ high VO_2max_ levels in our study, it is reasonable to say that physical fitness may affect cognition. On the other hand, there was no difference between athlete groups in terms of VO_2max_ levels. Our results showed a difference in cognitive functions, including visuospatial working memory and attention functions between athletes with similar VO_2max_ and physical activity levels from different sport categories. These findings may suggest that even when physical fitness levels are similar, differences may develop in cognition depending on exercise type.

The transfer of learning defines previous experiences’ effect on learning a new skill in a new context. Components of the skills such as their kinematics characteristics and/or the cognitive demands in which the skills are performed are similar; learning transfer may occur. It was demonstrated that interactive sport types had broader effects on processing speed and attention scores than self-paced sports [57]. Adults who juggle have shown a temporary and selective structural change in brain areas that are associated with the processing and storage of complex visual motion [58]. Our findings demonstrating superiority in visuospatial working memory, attention, and verbal fluency in the fencer group may be related more complex motor repertoire of fencing and transfer of such skills to cognitive tasks.

Muscle spindles carry signals for limb position sense. Because of fencing is a sport that involves complex movements and needs agility, it requires changes in temporal sequencing of muscle activation for more efficient movement, strategies of change in motor unit involvement, increased velocity of neural conduction, changes in frequency and degree of muscle innervation, and the ability to maintain rapid motor unit firing. The role of muscle spindles should be considered in fencing, where knowledge of the limb position during rapid movements is essential and scored by touching spesific areas of the opponent. Exercise-induced neuronal activation occurs via cerebral afferent stimulation through muscle spindle receptors [59]. No training mode can enhance the sensory receptor density, but it may increase the fusimotor drive to the muscle spindles in such challenging tasks and broaden the somatosensory area for proprioception in the sensory cortex. This mechanism may include learning to pay attention to a cue if it is important for performance. Increased selective attention to a cue creates changes in the primary sensory cortex [60], in summary, individuals become more skilled in processing sensory cues. Regarding that fencing is a sport performed with standing leaps and more position change in space, the sensory cortex area’s organization may become more dynamic due to the frequent stimulation of proprioceptive mechanoreceptors. This mechanism may explain the shorter reaction times for selective attention tasks in fencers in this study.

Environmental enrichment is one of the factor involved in the effect of exercise on cognition; a more challenging environment promotes more sensory, cognitive, and motor experiences. In fencing, the opponent’s unpredictable behavior and continuously shifting positions can be considered within the context of environmental enrichment. Fencers are exposed to more stimuli than swimmers. Therefore, the environmental enrichment factor in open-skill exercise may strengthen the exercise effect on cognition.

According to the broad transfer hypothesis, the long-term practice of specific skills can improve cognition for circumstances outside the specific sport context [61]. For example, fencing requires the inhibition of planned actions against the opponent’s tactical deceptions. Fencers have been shown to make fewer errors in tasks requiring inhibition than non-fencers. In this study, superior inhibitory control in visuospatial attention and verbal fluency tasks of fencers may provide evidence for the broad transfer hypothesis. It seems like sport expertise may be transferred from sports-specific contexts to general cognitive contexts.

Exercise activates signaling pathways at the cellular and molecular level to support brain plasticity [62]. Central myelination is a large part of plasticity, and different exercise modes stimulate different changes in motor circuits [63]. Cognitively demanding exercise provides neuronal survival in the hippocampus, strengthened connections in critical white matter pathways, and myelination in associated cortical brain regions [64]. Since fencing requires more cognitive demand, it may increase central myelination and this may have provided in shorter RTs in the attention test.

The cerebellum is well-established as an important subcortical brain region that plays a critical role in cognition and learning. It participates in voluntary shift of selective attention between sensory modalities and goal-directed cognitive functions [65, 66]. Complex motor skill training induces strengthening of a subset of parallel fiber synapses onto Purkinje cells and accompanies neuroplasticity [67]. Lunge and fleche are fast attacking techniques that need balance in fencing. Considering the role of the cerebellum in performing quick and precise movements. fencing may be a sport requiring more cerebellum involvement than swimming. Therefore, myelination may be observed more in fencers at the cerebellar level. Due to the cerebellum/lateral cerebellar nucleus’s dentate nucleus are crucial structures responsible for complex cognitive processing such as spatial navigation and working memory [68], fencers’ higher scores in attention and working memory tasks may be understood on the basis of cerebellar myelination.

In terms of human social cognition, the mirror neuron system has been reported to control the ability to understand others’ actions, communicate, imitate, and act in cooperation with others [69]. Because fencing is a more "social" sport than swimming with its direct contact with the opponent, understanding and making quick decisions about the opponent’s intention may activate the mirror neuron system. Higher scores of fencers in attention, working memory, and inhibition tasks may be linked to the mirror neuron system’s activation.

## Limitations

The present study had some limitations that need to be considered. The cross-sectional design simply reveals a possible relationship regarding how exercise type affects cognitive functions and peripheral proteins. Therefore, longitudinal studies are needed to show the causal relationship in the future.

Unequal branch variance in athlete groups was the other limitation. Although the fencing group included all branches of epee, sabre, and foil, the numbers were not equal, and in the swimming group, all were mid-distance swimmers except 2 short-distance participants. Also, we could not reach the number of participants we determined by assuming the type of study design and this limitation decreased relatively the effect sizes. In future studies, more participants in the same branches and expanded sample size will provide more reliable results. Lastly, the closed-skill sport we used in the study is performed in water. It would be more accurate to compare sports done on similar ground.

## Conclusion

The current study pointed out that different exercise-mode has different effects on cognition and proteins associated with cognition.

Because our data indicate that open-skill exercise stimulates and improves specific types of executive-control functioning, this exercise mode can be included in planning preventive and therapeutic exercises for cognitive loss.

Additionally, due to our results based on young adults, we anticipate that open-skill exercise might be improving not only elderly but also younger population. For instance, open-skill exercise in closed-skill athletes’ training programs may enhance their sports performance by adding cognitively demanding tasks.

To the best of our knowledge, this is the first study to assess serum CTSB level after an acute bout of exercise. The present findings clearly suggest that further research and evidence are required to understand the relationship between exercise, peripheral proteins and cognition.

## Supporting information

S1 Dataset Demographic and physical fitness data. (XLSX)

S2 Dataset Behavioral and blood parameters data. (XLSX)
